# Role of respiratory secretion culture in the surgical outcome prediction of bacterial empyema

**DOI:** 10.1007/s11748-025-02124-3

**Published:** 2025-02-01

**Authors:** Chia-Chi Liu, Ya-Fu Cheng, Yi-Ling Chen, Ching-Yuan Cheng, Chang-Lun Huang, Wei-Heng Hung, Bing-Yen Wang

**Affiliations:** 1 Division of Thoracic Surgery, Department of Surgery, Changhua Christian Hospital, Changhua City, Changhua County, Taiwan; 2 Surgery Clinical Research Center, Changhua Christian Hospital, Changhua City, Changhua County, Taiwan; 3 Department of Post-Baccalaureate Medicine, College of Medicine, National Chung Hsing University, Taichung City, Taiwan

**Keywords:** Bacterial empyema, Pleural effusion culture, Pleural tissue culture, Respiratory secretion culture, Surgical outcome

## Abstract

**Objectives:**

Thoracic empyema is a serious infection. Video-assisted thoracoscopic surgery is a recommended treatment, and pleural fluid and tissue cultures are collected intraoperatively. The combination of a pleural peels tissue culture and a pleural fluid culture improves the positive culture rate. We aimed to investigate the role of respiratory secretion cultures to determine the optimal management for improving surgical outcome.

**Methods:**

The study analyzed 225 adult patients with phase II/III thoracic empyema who underwent thoracoscopic decortication. Respiratory secretion cultures were obtained and compared with pleural cultures. Key outcomes were culture positivity and pathogen consistency, with secondary outcomes including intensive care unit stay, hospital stay, and mortality.

**Results:**

There were 225 empyema patients with either a positive pleural fluid culture or a positive pleural peel tissue culture. Of these, 76 patients had positive respiratory secretion culture findings during hospitalization. The most common pathogen species were *Pseudomonas aeruginosa* (44%) and *Klebsiella pneumoniae* (16%) in the respiratory secretion cultures and *Streptococcus* spp*.* (38%) and *Klebsiella pneumoniae* (12%) in the pleural cultures. There were 30 patients having a common pathogen in the respiratory secretion culture and in the pleural fluid/tissue culture. Poor outcome measures were found in these patients, including the longer use of antibiotics preoperatively [2.50 (1.00–6.00) days versus 5.00 (2.75–11.00) days, *p* = 0.006] and a higher mortality rate during hospitalization (40.0% versus 17.4%, *p* = 0.002).

**Conclusions:**

Respiratory secretion cultures are vital for predicting surgical outcomes in bacterial empyema, and prompt specimen collection can improve patient survival.

**Supplementary Information:**

The online version contains supplementary material available at 10.1007/s11748-025-02124-3.

## Objectives

Thoracic empyema is increasingly recognized as a serious infectious disease worldwide. Despite the application of advanced vaccine and antibiotics for prevention and treatment, the USA still reports approximately 60,000 patients annually, with an increasing incidence rate over the past decade [1, 2]. The adult mortality rate was about 20% in the 1990s, and recently it was around 8.7% for those who underwent thoracic decortication [2, 3].

According to the 2017 American Association for Thoracic Surgery guidelines, empyema can be classified into the early exudative phase (stage I), intermediate fibroproliferative phase (stage II), and organizing phase (stage III) [4]. Surgical intervention and chest tube drainage are not required for stage I empyema treatment [5]. For stage II and stage III empyema, closed drainage and decortication are strongly recommended. Recent studies revealed that video-assisted thoracoscopic surgery (VATS) provides similar surgical outcomes and fewer postoperative complications [6–10].

Bacterial pneumonia is the most common etiology of empyema, and 20 to 40% of bacterial pneumonia patients develop parapneumonic effusions that may progress to empyema [11]. Obtaining an accurate culture result early is essential for proper antibiotics usage in treating empyema or preventing it at the pneumonia stage. Previous literature report a pleural fluid culture-positive rate ranging from 19 to 49%, obtained through thoracentesis or intraoperatively [12, 13]. Combining pleural peels tissue culture and a pleural fluid culture, obtainable through VATS decortication, has been shown to improve the positive culture rate in empyema [14]. While respiratory secretion culture is the simplest and the most direct pathway to acquire pneumonia pathogens, its role in treating bacterial empyema has rarely been discussed in the literature. The relationship between respiratory secretion cultures and pleural fluid/tissue cultures also remains unknown.

This study aimed to investigate the consistency of respiratory secretion cultures and intraoperative pleural effusion/tissue cultures to determine the optimal management for improving surgical outcomes in bacterial empyema patients.

## Methods

### Patient population and selection

Informed consent from all participants was waived by the Changhua Christian Hospital, Changhua, Taiwan Institutional Review Board (IRB-220907). All methods were carried out in accordance with relevant guidelines and regulations. A search of the database of our institution (Changhua Christian Hospital, Changhua, Taiwan) identified 1197 patients over 18 years old with phase II or III thoracic empyema who underwent video-assisted thoracoscopic decortication of the pleura between April 2011 and May 2022. Exclusion criteria for the study were as follows: (1) the empyema patients’ chest X-ray or chest CT scan presented negative finding of pneumonia patch (*n* = 256); (2) patients had an operation without a positive finding from neither an intraoperative pleural fluid culture nor an intraoperative pleural peel tissue culture (*n* = 441); (3) patients who had no respiratory secretion culture during hospitalization (*n* = 194); (4) respiratory secretion culture yielded *Mycobacterium tuberculosis* or fungus (*n* = 43); (5) poor quality of respiratory secretion culture such as mixture with saliva or low specimen amount (*n* = 38). The remaining 225 patients were analyzed as subjects of this study.

### Obtaining the specimen

The presence of loculated pleural effusion in the patients’ chest image indicated a high suspicion of phase II/III empyema, suggesting the need for surgical intervention. We performed video-assisted thoracoscopic decortication and obtained both a pleural fluid culture and a pleural peel tissue culture intraoperatively. Respiratory secretion cultures were obtained one to four times from each patient through three methods: patient expectorating actively, aspiration by suction tube once the patient was under endotracheal tube intubation or with tracheostomy, and bronchoalveolar lavage (BAL). Both aerobic cultures and anaerobic cultures were done routinely for different specimens. For patients who expectorated respiratory secretions, oral hygiene could not be consistently controlled. However, for ICU patients with endotracheal intubation, nurses provided regular oral care using chlorhexidine gluconate daily. In patients who underwent bronchoscopy without an endotracheal tube, oral rinsing was performed prior to the procedure to minimize contamination from normal oral flora and reduce the risk of false-positive results.

### Clinical features and data collection

We analyzed the age, gender, empyema location, empyema phase, comorbidities, laboratory data, pleural fluid data, antibiotics usage, chest tube drainage duration, length of intensive care unit stay (ICLOS), length of hospital stay (HLOS), pathogen, and mortality. Two mortalities were analyzed: death during hospitalization and death within 30 postoperative days. All our observations were defined according to the 2017 American Association for Thoracic Surgery consensus guidelines. The indicators of infectious pleural fluid include pH value less than 7.2, glucose less than 40 mg/dL, and lactate dehydrogenase (LDH) greater than 1000 IU/L. The primary outcomes for our study were the respiratory secretion culture-positive rate and the consistency of pathogen between respiratory secretion culture and either pleural fluid culture or pleural peel tissue culture. The secondary outcomes were ICLOS, HLOS, mortality, and culture-positive rate by the method of specimen obtainment.

### Statistical analyses

Clinical factors including age, gender, empyema location, empyema phase, comorbidities, laboratory data, pleural fluid data, antibiotics usage, chest tube drainage duration, ICLOS, HLOS, pathogen, and mortality were analyzed. We used the Mann–Whitney *U* test to evaluate the continuous variables in this study. As for the categorical variables, we used the Chi-squared test to compare the proportions. A *p* value less than 0.05 was considered to indicate statistical significance. All statistical analyses were performed using SPSS statistical software (SPSS package, version 23.0; SPSS Inc., Chicago, IL, USA).

## Results

Two hundred and twenty-five stage II to stage III thoracic empyema patients, who underwent video-assisted thoracoscopic decortication and had a positive pleural fluid or pleural peel tissue culture, were included in this study. From these patients, one to four sets of respiratory secretion cultures were collected during the period of hospitalization. For 76 patients, at least one respiratory secretion culture set showed a positive finding, and 149 patients had only negative respiratory secretion culture results.

The clinical demographic data of the patients are shown in Table 1. The mean age was about 63 years, and most of the patients were males (75.6%). Right-side empyema was predominant (56.0%), and only one patient had bilateral empyema. There were a greater number of patients with stage II empyema (76.0%) than stage III empyema (24.0%). As for the comorbidities, only for congestive heart failure, the respiratory secretion culture-positive group had a statistically significantly higher ratio (15.8% versus 6.0%, *p* = 0.033). No differences in serum leukocyte count and pleural fluid analysis were noted between the two groups. Treatment outcome measures such as preoperative antibiotics use days, chest tube drainage duration, ICLOS, HLOS, and hospital mortality rate were statistically significantly lower in the respiratory secretion culture-negative group. There was no significantly higher 30-day mortality rate in the positive respiratory secretion culture cohort.

**Table 1:** Clinical characteristics of the patients

Factors, medians (IQR)	Total cohort (*n* = 225)	Culture positive (*n* = 76)	Culture negative (*n* = 149)	P value
Age, years	65.00 (52.00–76.00)	66.00 (56.00–76.00)	64.0 (49.5–76.0)	0.215
Gender				
Male	170 (75.6%)	54 (71.1%)	116 (77.9%)	0.566
Female	55 (24.4%)	22 (28.9%)	33 (22.1%)	0.327
Location				
Right	126 (56.0%)	40 (52.6%)	86 (57.7%)	0.635
Left	98 (43.6%)	35 (46.1%)	63 (42.3%)	0.670
Bilateral	1 (0.4%)	1 (1.3%)	0 (0.0%)	0.317
Phase				
II	171 (76.0%)	60 (78.9%)	111 (74.5%)	0.747
III	54 (24.0%)	16 (21.1%)	38 (25.5%)	0.466
Comorbidity				
Malignancy	61 (27.1%)	24 (31.6%)	37 (24.8%)	0.354
DM	82 (36.4%)	34 (44.7%)	48 (32.2%)	0.138
HTN	134 (59.6%)	51 (67.1%)	83 (55.7%)	0.321
Liver cirrhosis	19 (8.4%)	7 (9.2%)	12 (8.1%)	0.808
ESRD	25 (11.1%)	11 (14.5%)	14 (9.4%)	0.221
CHF	21 (9.3%)	12 (15.8%)	9 (6.0%)	0.033
COPD	54 (24.0%)	21 (27.6%)	33 (22.1%)	0.396
Laboratory data				
WBC (10^3^/μL)	14.60 (11.05–19.30)	13.30 (9.45–18.10)	14.90 (11.90–19.55)	0.075
ANC (10^3^/μL)	12.31 (9.10–16.55)	11.63 (7.66–16.30)	12.73 (9.73–16.61)	0.189
Pleural data				
Pleural pH (≦ 7.2)	112 (57.1%)	39 (59.1%)	73 (56.2%)	0.780
Pleural glucose (≦ 40 mg/dL)	112 (54.1%)	34 (48.6%)	78 (56.9%)	0.437
Pleural LDH (≧ 1000 IU/L)	152 (73.8%)	51 (72.9%)	101 (74.3%)	0.934
PreOP ABX usage (days)	3.00 (1.00–6.00)	4.00 (2.00–7.75)	2.00 (1.00–5.00)	0.014
PreOP chest tube drainage duration	0.00 (0.00–2.00)	0.00 (0.00–4.00)	0.00 (0.00–5.00)	0.143
PostOP chest tube drainage duration	10.00 (7.00–17.00)	13.00 (8.00–26.75)	9.00 (7.00–16.00)	< 0.001
Chest tube drainage duration	11.00 (7.00–19.00)	14.50 (9.00–28.00)	10.00 (7.00–16.00)	< 0.001
Length of ICU admission	7.00 (0.00–14.00)	13.00 (4.25–23.75)	4.00 (0.00–12.00)	< 0.001
Length of hospital stay	19.00 (12.00–32.00)	27.00 (17.00–44.75)	16.00 (11.00–30.00)	< 0.001
Hospital mortality	36 (16.0%)	20 (26.3%)	16 (10.7%)	0.014
Mortality in 30 days	19 (8.4%)	9 (11.8%)	10 (6.7%)	0.191

IQR: interquartile range; DM: diabetes mellitus; HTN: hypertension; ERDS: end-stage renal disease; CHF: congestive heart failure; COPD: chronic obstructive pulmonary disease; WBC: white blood cell; ANC: absolute neutrophil count; LDH: lactate dehydrogenase; OP: operative; ABX: antibiotics; ICU: intensive care unit

**Fig. 1:**
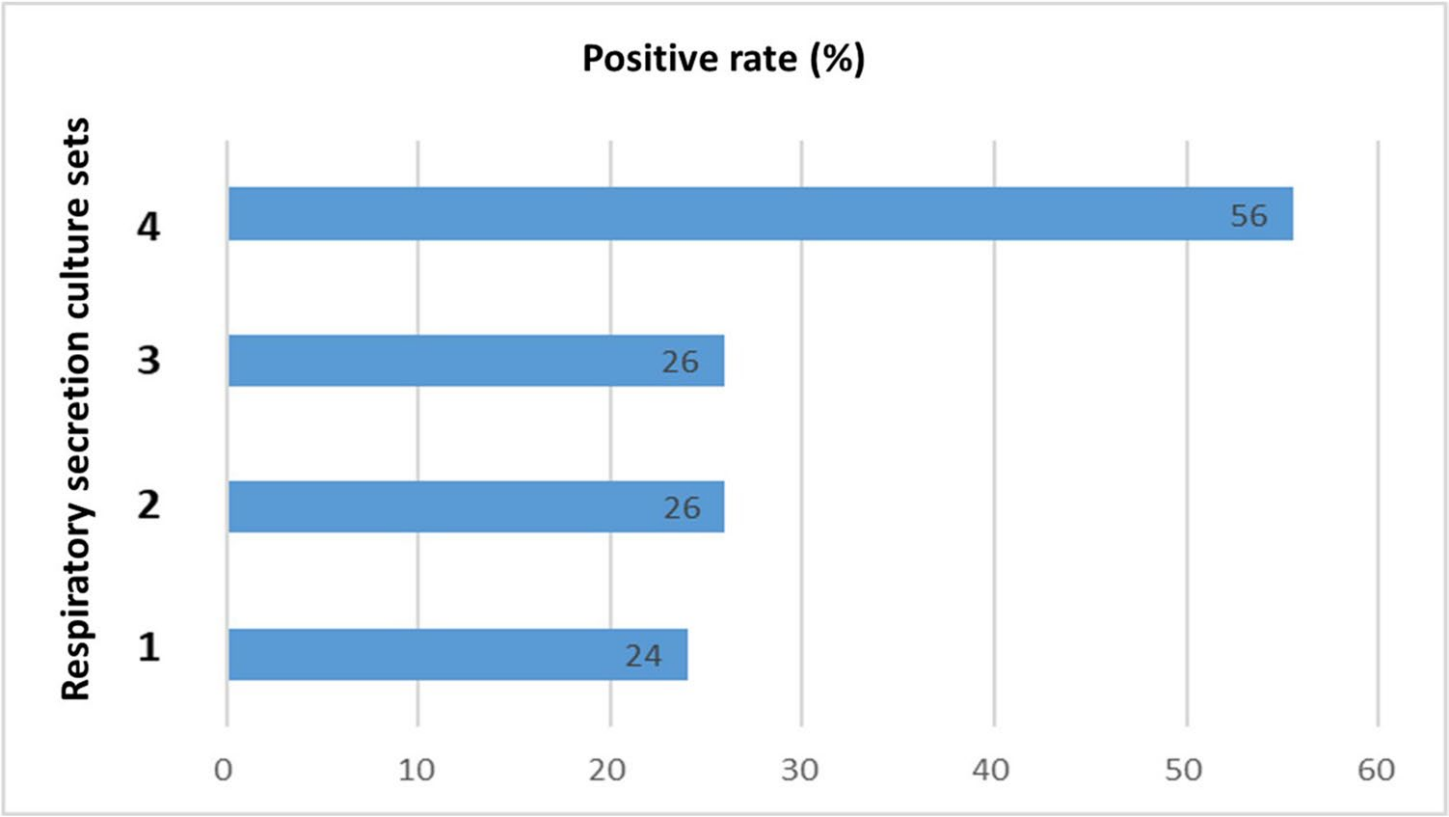
Positive culture rate by quantity of respiratory secretion cultures

We analyzed the patients based on the number of sets of respiratory secretion cultures (Fig. 1). Those with only one respiratory secretion culture set had a 24% positive rate, and similar positive culture rates were found in the patients with two sets (26%) and three sets (26%). In comparison, patients with four sets of respiratory secretion cultures had a significantly higher positive rate (56%).

**Fig. 2:**
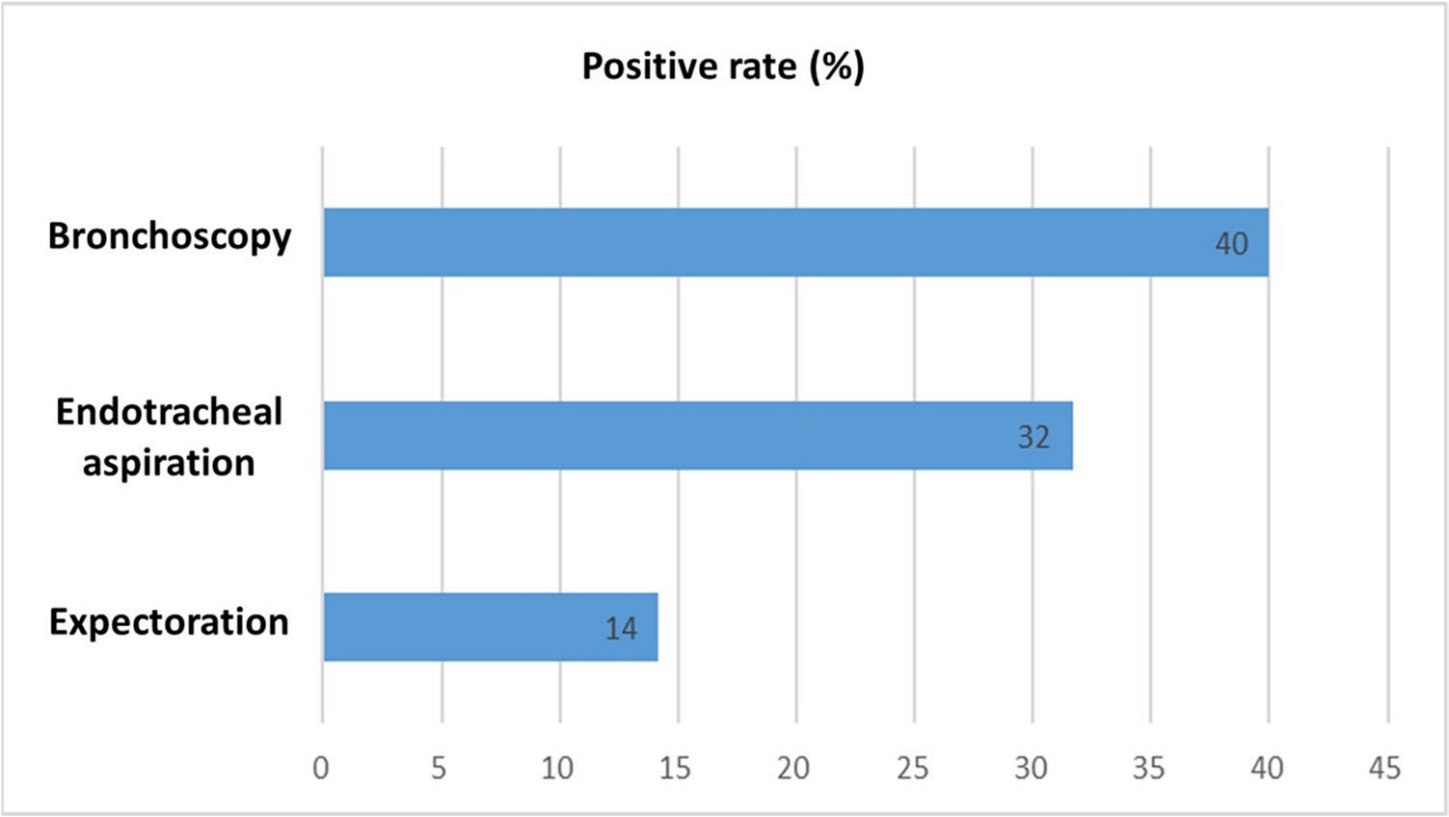
Positive culture rate by method of obtaining respiratory secretion specimens

The collection method of the respiratory secretion specimen was taken into consideration. In our center, we collected patients’ respiratory secretion in three ways: (1) patient expectorates by oneself, (2) aspiration through endotracheal tube or tracheostomy, (3) BAL under bronchoscopy. We analyzed the positive culture rates of these three methods separately (Fig. 2) and discovered significantly higher positive culture rates when the specimen was derived through bronchoscopy (40%) or aspiration via endotracheal tube or tracheostomy (32%).

**Fig. 3:**
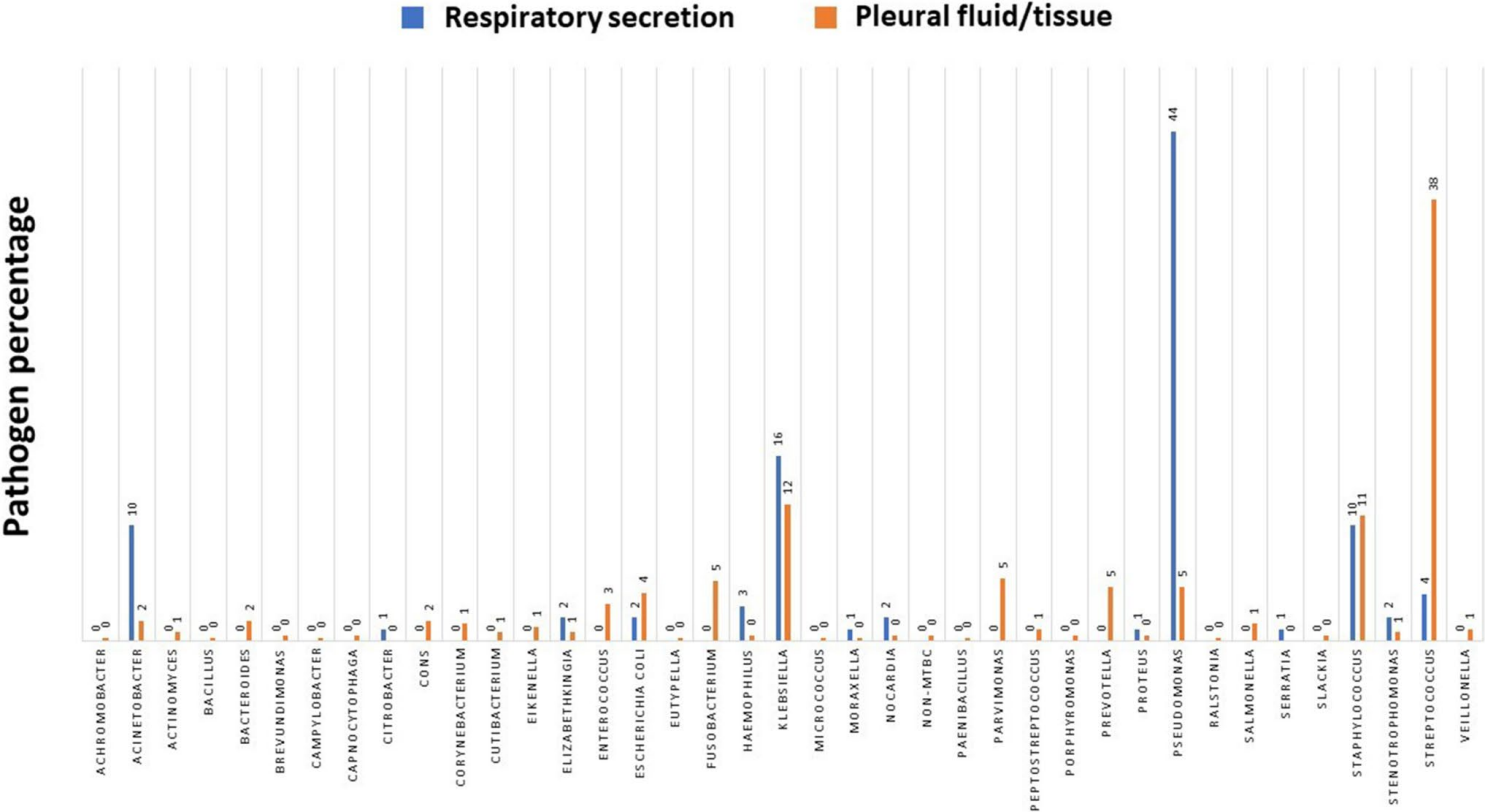
Pathogen distributions. *Pseudomonas aeruginosa* (44%), *Klebsiella pneumoniae* (16%), and *Staphylococcus aureus* (10%) were the most common pathogen species in the respiratory secretion cultures (blue bars). *Streptococcus* spp. (38%), *Klebsiella pneumoniae* (12%), and *Staphylococcus aureus* (11%) were the most common pathogen species in pleural effusion/tissue cultures (orange bars)

Figure 3 shows the pathogen distributions for the respiratory secretion cultures and the intraoperative pleural fluid and tissue peel cultures. In our study, the most common pathogen species in the respiratory secretion cultures was *Pseudomonas aeruginosa* (44%), followed by *Klebsiella pneumoniae* (16%), *Staphylococcus aureus* (10%), *Acinetobacter baumannii* (10%), and *Streptococcus* spp. (4%). The most common pathogen species in the pleural fluid cultures and pleural tissue peel cultures were *Streptococcus* spp. (20%), *Klebsiella pneumoniae* (16%), and *Staphylococcus aureus* (11%).

To determine the concordance rate of pathogens between the respiratory secretion cultures and the intraoperative pleural fluid/tissue peel cultures, we divided those patients with positive respiratory secretion cultures into an identical group and a distinct group (Table 2). We defined the identical group as the one in which the patients had a common pathogen in the respiratory secretion culture and pleural fluid/tissue culture (*n* = 30). The distinct group was defined as the one in which patients who had no pathogen in common between the sets of respiratory secretion culture and the pleural fluid/tissue culture (*n* = 46). On comparing these two groups, we noted that the age, gender, location and phase of empyema, laboratory data, and pleural analysis data were similar, but a longer use of antibiotics preoperatively was found in the identical group [2.50 (1.00–6.00) days versus 5.00 (2.75–11.00) days, *p* = 0.006]. For the surgical outcomes, the duration of chest tube drainage, ICLOS, and HLOS were longer in the identical group, but the differences were not statistically significant. However, the mortality rate during hospitalization was significantly higher in the identical group (40.0% versus 17.4%, *p* = 0.002).

**Table 2:** Concordance rate between species from respiratory secretion culture and pleural effusion or tissue culture (at least one pathogen is the same)

Factors, medians (IQR)	Distinct (*n* = 46)	Identical (*n* = 30)	*p* value
Age, years	66.00 (57.75–79.00)	65.00 (54.00–76.00)	0.527
Gender, male	32 (69.6%)	22 (73.3%)	0.723
Location, right	21 (45.7%)	19 (63.3%)	0.262
Phase, II	38 (82.6%)	22 (73.3%)	0.332
PreOP ABX usage (days)	2.50 (1.00–6.00)	5.00 (2.75–11.00)	0.006
Laboratory data			
WBC (10^3^/μL)	13.40 (9.70–18.28)	13.20 (9.03–18.30)	0.811
ANC (10^3^/μL)	12.00 (8.05–15.98)	11.10 (7.18–16.65)	0.983
Pleural data			
Pleural pH (≦ 7.2)	23 (54.8%)	16 (66.7%)	0.344
Pleural glucose (≦ 40 mg/dL)	21 (48.8%)	13 (48.1%)	0.955
Pleural LDH (≧ 1000 IU/L)	29 (67.4%)	22 (81.5%)	0.199
PreOP chest tube drainage duration	0.00 (0.00–2.00)	0.00 (0.00–5.00)	0.226
PostOP chest tube drainage duration	13.50 (8.00–25.00)	13.00 (7.75–30.00)	0.746
Chest tube drainage duration	14.00 (8.75–26.25)	17.00 (8.75–32.50)	0.602
ICU duration (day)	13.00 (7.75–22.25)	13.50 (3.00–31.25)	0.957
Hospital duration (day)	28.00 (19.00–44.25)	22.00 (14.00–48.00)	0.466
Hospital mortality	8 (17.4%)	12 (40.0%)	0.029
30-Day mortality	4 (8.7%)	5 (16.7%)	0.293

IQR: interquartile range; OP: operative; ABX: antibiotics; WBC: white blood cell; ANC: absolute neutrophil count; LDH: lactate dehydrogenase; ICU: intensive care unit

In addition, we performed univariable and multivariable analysis of the factors associated with in-hospital mortality as shown in Table 3. In the univariable analysis, we found that comorbidity of end-stage renal disease, the pH value of pleural effusion, positive respiratory secretion culture result, common pathogen between respiratory secretion and pleural effusion/peel cultures, the duration of chest tube drainage, ICLOS, and HLOS were the independent factors affecting intra-hospital mortality. Furthermore, we performed multivariable analysis and the results showed that those patients with older age, positive respiratory secretion culture, higher concordance rate between respiratory secretion and pleural effusion/peel cultures, and longer ICLOS would have higher intra-hospital mortality rate.

**Table 3:** Univariable and multivariable analyses of factors affecting hospital mortality

Variables	Univariable		Multivariable	
	Odds ratio	*p* value	Odds ratio	*p* value
Age, years	1.02 (1.00–1.05)	0.061	1.09 (1.03–1.16)	0.005
Gender				
Male	1		1	
Female	1.04 (0.45–2.36)	0.933	0.46 (0.10–2.13)	0.321
Location				
Right	1		1	
Left	0.83 (0.40–1.74)	0.627	0.82 (0.21–3.26)	0.779
Bilateral	8.08 × 10^9^	> 0.999	4.64 × 10^10^	> 0.999
Phase				
II	1		1	
III	1.75 (0.81–3.79)	0.156	1.21 (0.24–6.14)	0.818
Comorbidity				
Malignancy	1.92 (0.91–4.06)	0.086	1.72 (0.61–4.84)	0.478
DM	1.95 (0.95–4.01)	0.068	1.63 (0.42–6.24)	0.544
HTN	1.44 (0.68–3.04)	0.345	1.46 (0.36–6.93)	0.602
Liver cirrhosis	2.02 (0.68–6.00)	0.207	6.14 (1.04–36.40)	0.046
ESRD	6.77 (2.77–16.53)	< 0.001	5.36 (1.24–23.14)	0.025
CHF	1.27 (0.40–4.01)	0.69	1.02 (0.15–7.12)	0.981
COPD	0.73 (0.30–1.77)	0.486	0.05 (0.01–0.52)	0.012
Laboratory data				
WBC, mean ± SD (10^3^/μL)	0.97 (0.91–1.03)	0.253	1.17 (0.70–1.97)	0.549
ANC, mean ± SD (10^3^/μL)	0.97 (0.91–1.03)	0.353	0.81 (0.46–1.40)	0.445
Pleural data				
Pleural pH (≦ 7.2)	2.59 (1.10–6.10)	0.029	3.73 (0.86–16.10)	0.079
Pleural glucose (≦ 40 mg/dL)	1.26 (0.60–2.65)	0.547	1.55 (0.30–1.20)	0.605
Pleural LDH (≧ 1000 IU/L)	1.13 (0.48–2.69)	0.779	0.18 (0.03–1.20)	0.076
Positive culture				
Pleural effusion	1.33 (0.55–3.24)	0.527	0.79 (0.13–4.71)	0.799
Tissue	1.39 (0.66–2.90)	0.387	2.64 (0.68–10.33)	0.163
Respiratory secretion	2.97 (1.43–6.15)	0.003	0.11 (0.01–0.88)	0.037
Culture concordance rate	4.75 (2.04–11.07)	< 0.001	56.79 (5.29–609.82)	0.001
Chest tube drainage duration	1.03 (1.01–1.04)	0.003	1.02 (0.98–1.06)	0.286
Length of ICU admission	1.08 (1.05–1.12)	< 0.001	1.16 (1.06–1.27)	0.001
Length of hospital stay	1.04 (1.02–1.06)	< 0.001	0.99 (0.94–1.04)	0.555

DM: diabetes mellitus; HTN: hypertension; ERDS: end-stage renal disease; CHF: congestive heart failure; COPD: chronic obstructive pulmonary disease; WBC: white blood cell; ANC: absolute neutrophil count; LDH: lactate dehydrogenase; ICU: intensive care unit

We conducted both univariable and multivariable analyses to assess the association between specific pathogens and in-hospital mortality. In the univariable analysis, *Pseudomonas aeruginosa* was identified as an independent factor affecting in-hospital mortality. However, in the multivariable analysis, none of the common pathogens, including *Pseudomonas aeruginosa*, *Klebsiella pneumoniae*, and *Staphylococcus aureus*, or other bacterial species, were found to be significantly linked to higher in-hospital mortality rates. Therefore, in our study, the presence of different bacterial species in respiratory secretions was not significantly associated with in-hospital mortality.

In addition, we performed a subset analysis of 64 patients whose respiratory secretions were obtained prior to initiating antimicrobial therapy (Supplement Table 1). Of these, 11 patients (17.2%) had positive respiratory secretion cultures, and 53 (82.8%) had negative cultures. Although the culture-positive group showed a significantly longer ICU stay (median 13.00 vs. 6.00 days, *p* = 0.037), the difference in overall hospital stay between the two groups did not reach statistical significance (median 36.00 vs. 15.00 days, *p* = 0.125). Furthermore, the hospital mortality rate was higher in the culture-positive group (18.2% vs. 7.5%), but this difference was not statistically significant (*p* = 0.271).

## Discussion

In this retrospective study, surgical outcomes were poor, with a higher in-hospital mortality rate observed among patients with a common pathogen in the intraoperative pleural fluid/tissue culture and a respiratory secretion culture set. After confirming the diagnosis of stage II to stage III thoracic empyema, decortication was performed, and pleural fluid and tissue cultures were obtained intraoperatively. Additionally, respiratory secretion cultures were obtained if recurrence or progression of the pulmonary infection was suspected, and antibiotic use was adjusted accordingly. To explain the higher in-hospital mortality in the identical pathogen group, the consistency between a patient’s respiratory secretion culture and intraoperative pleural fluid/tissue culture may imply a larger bacterial colony, the development of drug resistance, the inefficacy of antibiotics treatment, or inadequate length of therapy. It is essential for surgeons to recognize this result to consider the causes for improving surgical outcomes.

A previous study revealed that approximately 20% of community-acquired pneumonia cases developed parapneumonic effusions, and only 1.4% of those met the criteria of empyema [15]. The upper airway appears to be the most common route of pathogen invasion, and the cause of secondary bacterial invasion to the pleural space is not completely understood. Several studies suggest that pleural infection can occur spontaneously even in the absence of underlying lung consolidation [16–18]. This finding implies that there is another route causing infection in the pleural space. The possible mechanisms include visceral pleural defects or fistula formation, dispersion from the mediastinum, transdiaphragmatic infection, hematogenous spread, and trauma or iatrogenic injury [19]. In our data, 195 out of 225 patients had completely different pathogens cultured from respiratory secretion specimens and the pleural space.

The focus of this study was to evaluate the impact of respiratory secretion cultures and pleural fluid and peel tissue cultures on the surgical outcomes of empyema patients. Therefore, we initially excluded patients without positive findings from both pleural fluid and peel tissue cultures. Preliminary analysis of the surgical outcomes for these patients (*n* = 441) indicated more favorable results compared to those with positive pleural fluid or peel tissue cultures. Specifically, the average ICU admission duration was 1.05 days (median: 0 days), average hospital admission was 20.72 days (median: 15 days), and in-hospital mortality was 9.0% (40/441).

Based on the positive interoperative pleural effusion or tissue culture findings, we differentiated the methods of obtaining respiratory secretion cultures to determine the most effective one for pathogen identification. In an early research, Fabrice et al. investigated the role of routine endotracheal aspiration respiratory secretion culture on ventilator-associated pneumonia (VAP) patients. They compared endotracheal aspiration culture results to positive BAL cultures and found an 82% identical rate among the patients with less than 5 days of mechanical ventilation and an 86% identical rate among those with more than 5 days of mechanical ventilation [20]. This suggests that VAP can be detected sooner, allowing for the prescription of adequate antibiotics for treatment. However, Johannes et al. recently reviewed diagnostic studies of endotracheal aspiration and BAL culture methods for VAP and concluded in favor of BAL analysis due to the poor positive predictive value of endotracheal aspiration cultures [21]. In our research, the positive rate of BAL cultures is higher than that of endotracheal aspiration cultures (40% versus 32%), and the comparison of concordance rates to the pleural cultures showed an opposite result that was not statistically significant (25% versus 37%, *p* = 0.346). Both endotracheal aspiration and BAL culture methods are effective in antibiotic therapy adjustment and surgical outcome prediction in empyema patients.

The pathogen spectrum varies geographically and in terms of the timing of cultures. In a major review of microbiology of pleural infection by Hassan et al., *Staphylococcus aureus* was reported to be the most common organism worldwide, with pneumococci and viridans streptococci being the most common organism in the tropical regions [22]. The cases we collected were all from Taiwan, a tropical island, and in our study, the most common organism causing pleural infection was *Streptococcus* species, which was compatible with the geographical trends reported in the review.

Hospital-acquired pneumonia (HAP) is defined as pneumonia that develops 48 h or more after hospital admission and was not present or incubated at the time of admission. Among the various pathogens responsible for HAP, *Pseudomonas aeruginosa* is notably prevalent, particularly in high-risk populations including ICU patients, those with chronic underlying conditions, and individuals subjected to prolonged antibiotic therapy [23, 24]. In our study, *Pseudomonas aeruginosa* was isolated from 44 respiratory secretion cultures obtained from 39 patients. Notably, all sputum samples were collected more than 48 h after hospital admission, adhering to the established criteria for HAP. Consequently, the detection of *Pseudomonas aeruginosa* in these cultures unequivocally classifies these infections as hospital acquired. This finding underscores the significance of *Pseudomonas aeruginosa* as a prevalent pathogen in HAP cases within our hospital setting.

We emphasized empyema related to bacterial pneumonia and excluded the patients with fungal or tuberculosis empyema. Numerous studies have highlighted that the primary causes of community-acquired pneumonia were *Streptococcus* species and anaerobes, while HAP was mostly caused by *Staphylococcus aureus*. [25, 26] Major pathogens of VAP were reported to be *Pseudomonas aeruginosa*, *Klebsiella pneumoniae*, and methicillin-resistant *Staphylococcus aureus* (MRSA) [27, 28]. Our data indicated that the primary pathogens in respiratory secretion cultures were *Pseudomonas aeruginosa* (44%) and *Klebsiella pneumoniae* (16%), while the main pathogens in pleural effusion/tissue cultures were *Streptococcus* species (38%), *Klebsiella pneumoniae* (11%), and *Staphylococcus aureus* (10%). We performed VATS decortication soon after empyema was diagnosed or when it was suspected, usually within 7 days. Intraoperative pleural cultures could be obtained early in the hospital course. However, 61.8% of our patients underwent ICU admission and received endotracheal tube intubation with ventilator for oxygen support. Respiratory secretion cultures could be collected at any time during the hospital course. Notably, the specimens obtained by endotracheal aspiration or BAL would be highly associated with VAP, potentially explaining our observed pathogen spectrum.

In this study, 21 patients experienced postoperative chest tube drainage lasting more than 30 days. Of these, 13 patients were diagnosed with bronchopleural fistula formation with persistent air leakage, and all required ventilator support. This bronchopleural fistula formation could be a complication of VATS decortication, likely resulting from lung parenchymal injury sustained during the procedure, which could lead to persistent air leakage if the lung laceration did not heal adequately. The remaining eight patients had persistent purulent effusion, which we attributed to the recurrence of empyema rather than a failure of VATS decortication.

It is essential to start the treatment for empyema with broad-spectrum antibiotics initially [4]. Additionally, appropriately escalating the antibiotics regimen with anti-*Pseudomonas* or anti-MRSA agents based on the respiratory secretion culture result is crucial in both adult and pediatric patients [29, 30]. In our center, we initially prescribed empiric anti-*Pseudomonas* antibiotics when the empyema patients were admitted to ICU. Simultaneously, respiratory secretion and blood cultures were repeatedly collected until the infection was well under control. Further use of therapeutic anti-MRSA agent would be indicated based on the culture results. A high rate of positive respiratory secretion cultures (79 out of 137) was obtained after antimicrobial therapy, which suggests that bacterial substitution may have occurred in both the respiratory tract and the empyema cavity, potentially contributing to persistent infection and higher mortality rates. Indeed, prolonged antimicrobial use in more severe cases could allow certain bacteria to persist or become dominant despite therapy. Regarding the respiratory secretions obtained before antimicrobial therapy, our findings suggest that while early culture positivity before antimicrobial therapy may indicate a more complicated clinical course, it did not translate into significantly increased mortality in this subset. However, the limited number of patients in this analysis warrants caution, and further studies with larger samples are needed to validate these observations. Moreover, these findings indirectly illustrate why obtaining multiple culture sets can enhance the diagnostic accuracy. They also underscore the importance of early surgical intervention to collect diverse specimens, thereby facilitating the selection of appropriate antibiotic therapy.

Our study has several limitations. Firstly, the data are derived from a single medical center. The pathogen spectrum changes geographically, and the evidence is insufficient to be universally applicable. Secondly, this is a retrospective study, which may involve selection bias. Thirdly, the culture techniques vary among different institutes, potentially affecting sensitivity. A larger database or a multicenter study is needed to confirm our findings. Fourthly, the differences between respiratory secretion and pleural results may be due to the presence of two different and subsequent infections. The lack of data about infection source may cause potential bias. Fifthly, the administration of antibiotics prior to collecting respiratory secretion samples may have led to bacterial substitution, potentially affecting the pathogen distribution and introducing bias. This factor may have influenced our findings regarding the pathogen profile and matching between respiratory secretions and empyema cultures. Lastly, the timing to obtain respiratory secretion specimens during hospitalization was not regular. The number of cultures and the collection methods varied among different cases. Despite the limitations, respiratory secretion culture plays a crucial role in the prediction of surgical outcomes in bacterial empyema patients.

## Conclusion

In conclusion, our findings suggested that a positive respiratory secretion culture and the concordance between respiratory secretion culture and pleural fluid or tissue culture are associated with a higher hospital mortality rate. The prompt acquisition of a respiratory secretion specimen and early surgical intervention emerge as crucial factors for improving the survival rate and achieving better surgical outcomes for thoracic empyema patients.

## Supplementary Information

Below is the link to the electronic supplementary material.

Supplementary file1 (DOCX 23 KB)

## Data Availability

All relevant data supporting the findings of this study are available from the corresponding author and coauthors upon reasonable request, subject to institutional review board approvals and patient confidentiality protocols.
