# Supplementary material for: Role of respiratory secretion culture in the surgical outcome prediction of bacterial empyema
Source: Gen Thorac Cardiovasc Surg. 2025 Feb 1;73(9):682–91. doi: 10.1007/s11748-025-02124-3 (PMC12378877; doi:10.1007/s11748-025-02124-3)
Supplement: Supplementary file 1 — Supplementary file1 (DOCX 23 KB) [file 11748_2025_2124_MOESM1_ESM.docx]

Supplement table 1. Clinical outcomes in patients whose respiratory secretions were obtained before the initiation of antimicrobial therapy

| Factors, Medians (IQR) | Total cohort (n=64) | Culture positive (n=11) | Culture negative (n=53) | P value |
| --- | --- | --- | --- | --- |
| Length of ICU admission | 6.00(0.00-11.75) | 13.00(0.00-22.00) | 6.00(0.00-10.50) | 0.037 |
| Length of hospital stay | 18.00(10.25-31.75) | 36.00(10.00-60.00) | 15.00(10.50-28.50) | 0.125 |
| Hospital mortality | 6(9.4%) | 2(18.2%) | 4(7.5%) | 0.271 |
| Mortality in 30 days | 1(1.6%) | 0(0.0%) | 1(1.9%) | >0.999 |
| IQR: interquartile range; ICU: intensive care unit | | | | |
